# Immune Response and Partial Protection against Heterologous Foot-and-Mouth Disease Virus Induced by Dendrimer Peptides in Cattle

**DOI:** 10.1155/2018/3497401

**Published:** 2018-04-18

**Authors:** I. Soria, V. Quattrocchi, C. Langellotti, M. Pérez-Filgueira, J. Pega, V. Gnazzo, S. Romera, J. Schammas, D. Bucafusco, S. Di Giacomo, Beatriz G. de la Torre, D. Andreu, F. Sobrino, E. Blanco, P. Zamorano

**Affiliations:** ^1^Instituto de Virología, Centro de Investigaciones en Ciencias Veterinarias, Instituto Nacional de Tecnología Agropecuaria (INTA)-Castelar, Buenos Aires, Argentina; ^2^Consejo Nacional de Investigaciones Científicas y Técnicas (CONICET), Buenos Aires, Argentina; ^3^Departament de Ciencies Experimentals i de la Salut, Universitat Pompeu Fabra, 08003 Barcelona, Spain; ^4^Centro de Biología Molecular Severo Ochoa (CSIC-UAM), 28049 Madrid, Spain; ^5^Centro de Investigación en Sanidad Animal (CISA-INIA), Valdeolmos, 28130 Madrid, Spain; ^6^Universidad del Salvador, Buenos Aires, Argentina

## Abstract

Synthetic peptides mimicking protective B- and T-cell epitopes are good candidates for safer, more effective FMD vaccines. Nevertheless, previous studies of immunization with linear peptides showed that they failed to induce solid protection in cattle. Dendrimeric peptides displaying two or four copies of a peptide corresponding to the B-cell epitope VP1 [136–154] of type O FMDV (O/UKG/11/2001) linked through thioether bonds to a single copy of the T-cell epitope 3A [21–35] (termed B_2_T and B_4_T, resp.) afforded protection in vaccinated pigs. In this work, we show that dendrimeric peptides B_2_T and B_4_T can elicit specific humoral responses in cattle and confer partial protection against the challenge with a heterologous type O virus (O1/Campos/Bra/58). This protective response correlated with the induction of specific T-cells as well as with an anamnestic antibody response upon virus challenge, as shown by the detection of virus-specific antibody-secreting cells (ASC) in lymphoid tissues distal from the inoculation point.

## 1. Background

The foot-and-mouth-disease virus (FMDV) causes a highly contagious disease with high morbidity in cloven-hoofed animals, including cattle and swine. FMDV can be controlled by the use of a chemically inactivated whole-virus vaccine; however, some disadvantages are associated with the use of inactivated vaccine. For example, the vaccine provides short-term protection, resulting in the need for revaccination [1], and there is a risk of the infectious virus being released during vaccine production. Therefore, a number of countries with large livestock industries have abandoned vaccination. However, this policy leaves livestock herds prone to sudden outbreaks of FMD, with dramatic effects on livestock economy and animal welfare, as seen in the United Kingdom in 2001 [2, 3] and in turn has led to intensive research on alternative vaccination strategies.

The FMD viral particle consists of a positive-strand RNA genome, a single open reading frame (ORF) which encodes four capsid proteins, VP1, VP2, VP3, and VP4, and eleven different mature nonstructural proteins (NSP).

The B-cell binding site located in the G-H loop (around residues 140–160) of FMDV VP1 protein has been identified as a predominant epitope that elicits neutralizing antibodies against this virus in natural hosts and animal models [4, 5]. A T-cell epitope, located at residues 21 to 35 of FMDV NSP 3A, efficiently stimulates lymphocytes from pigs infected with a type C virus [6].

The current inactivated FMD vaccines only promote serological protection against a given FMDV serotype, do not confer interserotype protection, and may not, in some cases, confer intraserotype protection given the antigenic variation existing within some serotypes [7]. Additionally, these vaccines present other shortcomings, such as possible incomplete inactivation of virus, need for biosafety level 4 (BSL-4 OIE) laboratories, and requirement for a cold chain to preserve virus stability. On the other hand, the vaccine virus must be purified enough as not to induce detectable antibodies against viral NSP to allow a distinction between vaccinated and infected animals [8].

Peptide vaccines are an attractive alternative strategy that relies on the usage of short peptide fragments to engineer the induction of highly targeted immune responses, consequently avoiding allergenic and/or reactogenic sequences [9]. Various synthetic peptide or recombinant protein vaccines based on the FMDV VP1 G-H loop have been shown effective in pigs [10–12], but they have shown limited efficacy in cattle [13–15], pointing to the limitations of these vaccines in eliciting broad protective responses in different hosts. Synthetic peptides are particularly attractive FMDV vaccine candidates as they are highly pure, defined, stable, and safe, and due to their modular approach, they can incorporate different B- and T-cell peptides [9, 16].

Multiple antigenic peptides (MAPs) are dendrimeric (branched) macromolecules built from a lysine core from which a defined number of epitopes radiate [17, 18]. An effective peptide vaccine needs a B-cell epitope to elicit a high neutralizing antibody response and a T-cell epitope to provide adequate cooperation between T-cells and B-lymphocytes.

The dendrimeric peptide design improves the effectiveness of viral antigenic site presentation to the immune system. Recent studies indicate that vaccination with dendrimeric peptides based on the amino acid sequence of 3A (T-cell epitope) and VP1 GH loop (B-cell epitope) from the type O FMDV O/UKG/11/2001, and branched by means of thioether or maleimide conjugation chemistries, elicits an immune response that achieved protection in up to 100% of the vaccinated pigs [16]. Likewise, we recently reported that similar dendrimeric peptides, based on the amino acid sequences from the type O FMDV O1/Campos/Bra/58, including a VP4 sequence as T-cell epitope, can protect cattle against homologous challenge [19].

The aim of this study was to investigate whether dendrimeric peptides elicited protection against heterologous viruses, a relevant issue for efficient vaccine design. To this end, the immune response elicited in cattle by dendrimers containing amino acid sequences of 3A and VP1 GH loop from type O FMDV O/UKG/11/2001, B_2_T and B_4_T, and the protection they afforded against the heterologous type O virus O1/Campos/Bra/58, was analyzed.

Our results indicate that B_2_T and B_4_T elicited specific humoral responses in cattle and conferred partial protection against the challenge with a heterologous virus O1/Campos/Bra/58. This protective response correlated with the induction of FMDV-specific T-cells as well as with an anamnestic antibody response upon virus challenge, as shown by the detection of virus-specific ASC in lymphoid tissues distal from the inoculation point.

## 2. Material and Methods

### 2.1. Peptides

**Figure 1:**
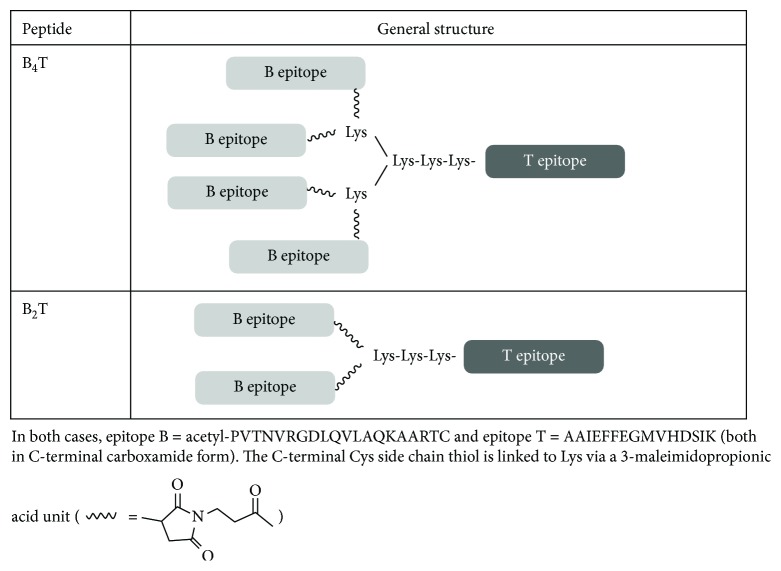
Dendrimeric peptides used in this study.

The dendrimeric peptides reproduced the B-cell (PVTNVRGDLQVLAQKAART, residues 136–154 of VP1) and T-cell (AAIEFFEGMVHDSIK, residues 21–35 of 3A) epitopes of FMDV O-UKG 11/01 (Figure 1). As detailed in [19], B_2_T and B_4_T constructions were assembled by conjugation of a T-epitope N terminally elongated with Lys residues providing 2 or 4 levels of branching and functionalized with chloroacetyl units and an N-acetylated B epitope with a C-terminal Cys whose thiol group reacts with the chloroacetyl group to give a thioether link. Additional details on the synthesis are available in previously published works [11, 20]. The final products were purified to near homogeneity by HPLC and characterized by mass spectrometry.

### 2.2. Virus

FMDV O1/Campos/Bra/58 was kindly provided by Biogenesis Bagó SA as binary ethylene-imine (BEI) inactivated (iFMDV). Purified virus was obtained by a sucrose density gradient centrifugation method [21] and was used for ELISA and lymphoproliferation assay. For challenging and virus neutralization assays, infective FMDV O1/Campos/Bra/58 (kindly donated by the Argentine National Service of Animal Health) was used in BSL-4 OIE laboratories and boxes at INTA. The sequence corresponding to the B-cell epitope of VP1 from FMDV O1/Campos/Bra/58 (140–158) comprises the amino acid residues ***A***V***P***NVRGDLQVLAQK***V***ART. The amino acids that differ between strains O1/Campos/Bra/58 and O/UKG/11/2001 are those corresponding to positions 140, 142, and 156 (indicated with the italic and bold formats).

A virus stock derived from FMDV isolated O/UKG/11/2001 (The Pirbright Institute, UK) by two amplifications in swine kidney cells was used in the virus neutralization assays.

### 2.3. Animals, Vaccines, Immunization, Infection, and Sampling of Cattle

Ten Hereford calves serologically negative for FMDV, approximately 6 months old, were used in the experiment. Groups of four animals were inoculated twice (days 0 and 18), by subcutaneous injection in the front left quarter, with 2 mg of B_2_T or B_4_T peptide in 2 ml of a water-in-oil single emulsion. The adjuvant included was the same contained in commercial vaccines. At 38 days postvaccination (dpv), the animals were challenged by nasal instillation with 1 ml (0.5 ml for each nostril) of 10000 of 50% bovine infective doses (BID50) of infective FMDV O1/Campos/Bra/58 (determined by titration on cattle tongue) [22–26]. This method is intended to mimic a natural FMDV infection [27]. Control unvaccinated bovines (*n* = 2) were challenged at the same time, and the same procedure was followed. All animals were monitored for 7 days for the emergence of FMD clinical signs and then were euthanized. The clinical score was determined by the number of feet presenting FMD lesions (with score one for each foot with lesions typical of FMDV) plus the presence of vesicles in the snout (score one) and/or mouth (score one), 6 being the maximum score.

Seven days postchallenge (dpc), all animals were checked for FMDV-induced lesions on the feet and tongue. Bovines with the absence of FMDV-induced lesions at the feet were considered as protected to podal generalization (PPG), while, animals with a delay in the onset of symptoms were considered partially protected (PP). At 7 dpc, different lymphoid organs were obtained postmortem from each animal: mandibular lymph nodes (ML), medial retropharyngeal lymph nodes (MRL), and tracheobronchial lymph nodes (TBL). All lymphoid organs were collected aseptically and placed in ice-cold wash buffer (RPMI 1640, 10 mM HEPES, 100 U/ml penicillin G sodium, 100 *μ*g/ml streptomycin, and 20 *μ*g/ml gentamicin) until processing.

Another five calves were immunized by subcutaneous injection with a single dose of commercial FMDV vaccine (water-in-oil single emulsion containing FMDV strains A Arg 2000, A Arg 2001, A24 Cruzeiro, and O1 Campos). This vaccine has been approved by the Argentine Animal Health Service (SENASA) with more than 80% of expected percentage of protection against all vaccine strains [28]. Experiments were performed according to the INTA ethics manual *Guide for the Use and Care of Experimental Animals*. The protocol was approved by the Institutional Animal Care and Use Committee (CICUAE INTA CICVyA) (Permit Number: 14/2011).

### 2.4. Measurement of Anti-Dendrimer and Anti-FMDV Antibodies

For the estimation of the immune response elicited by the dendrimers, we followed the methods of Soria et al. [19]. An indirect ELISA was used for anti-dendrimer antibody measurement. MaxiSorp 96-well plates (Nunc) were coated with B_4_T peptide (30 *μ*g/ml), the plates were washed and blocked with PBST-OVA 1%, and dilutions of serum samples were added. After incubation, the plates were washed and horseradish peroxidase- (HRP-) labeled goat anti-bovine IgG antibody (KPL, USA) was added. After washing, *ortho*-phenylenediamine- (OPD-) H_2_O_2_ was added as HRP substrate.

FMDV-specific antibodies were detected by means of an indirect ELISA, as described by Quattrocchi et al. [29]. Briefly, Immulon II 96-well ELISA plates were coated with 2.6 *μ*g/ml FMDV O1/Campos/Bra/58 and processed as described above.

The antiviral ELISA detailed above was modified in order to detect FMDV-specific IgG1 and IgG2 (in sera) and IgG1 and IgA (in nasal swabs) antibodies. After incubation with samples, a mouse anti-bovine IgG1, IgG2, or IgA monoclonal antibody was added (kindly provided by Dr. S. Srikumaran, University of Nebraska, USA). Lastly, a (HRP)-labeled goat anti-mouse IgG antibody was added after wash. OPD was used as HRP substrate. Absorbance was recorded at 492 nm (A492) in a microplate photometer (Multiskan FC, Thermo). The cut-off was established as the mean A492 of the negative sera (from all unvaccinated animals) plus two standard deviations (SD). Antibody titres were calculated for IgG1 and IgG2 as log_10_ of the last reciprocal dilution above cut-off. IgA levels were expressed as the ratio between the OD A492 of the nasal swabs from 22 dpv to 0 dpv. Positive control sera were included in every plate.

### 2.5. Neutralizing Index

The neutralizing index (NI) of serum (variable virus and fixed serum) from cattle immunized with B_2_T, B_4_T, or conventional vaccine, at 38 dpv (upon 2 doses of peptide), was measured. A 1/16 serum dilution was incubated with 10-fold dilutions of infective FMDV (1000 to 1 of 50% tissue culture infective dose—TCID50), and the infective virus recovered was determined by a TCID50 assay. The NI of a serum was calculated as the ratio between the titres of the virus in the presence of vaccinated animal serum and in the presence of a negative serum. The results were expressed as log_10_ of NI.

### 2.6. Neutralizing Antibody Titres

Serum samples were examined for anti-FMDV neutralizing antibodies (fixed virus and variable serum) as described before [29]. Briefly, serial dilutions (from 1/4 to 1/512) of inactivated sera were incubated for 1 h at 37°C with 100 TCID50 of infective FMDV O/UKG/11/2001 or O1/Campos/Bra/58. Then virus-serum mixtures were seed on BHK-21 monolayers. After 40 min at 37°C, fresh MEM-D/2% fetal calf serum was added to the monolayers and incubated at 37°C, under 5% CO_2_. Cytopathic effects were observed after 48 h. Titres of virus neutralizing antibodies (VNT) were expressed as log_10_ of the reciprocal of the serum dilution, which neutralizes 50% of 100 TCID50 FMDV.

### 2.7. Lymphoproliferation Assay

Peripheral blood mononuclear cells (PBMC) were obtained from cattle as described [30]. To this end, 100 *μ*l of 2.5 × 10^6^ cells/ml suspension were added to 96-well plate containing (i) 5 *μ*g/ml iFMDV; (ii) 50 *μ*g/ml of B_2_T, B_4_T, or T peptides; and (iii) 5 *μ*g/ml concanavalin A (Sigma-Aldrich, St. Louis, MO) and the cells were incubated at 37°C in 5% CO_2_ atmosphere for 4 days. During the last 18 h of culture, 1 *μ*Ci [3H]-thymidine (sp. act. 20 Ci (740 Gbq)/mMol; PerkinElmer) was added to each well. Cells were collected using a semiautomatic harvester (Skatron), and the incorporation of radioactivity into the DNA was measured by liquid scintillation counting with a counter unit (Wallac 1414, PerkinElmer) that was controlled by the WinSpectral software system. Results were expressed as stimulation index (SI). The SI was calculated as the cpm of antigen-specific proliferation/cpm of cell basal proliferation (in the absence of antigen).

### 2.8. Interferon-Gamma Detection

PBMC were cultured with either 50 *μ*g/ml of B_2_T, B_4_T, or T peptides or with 5 *μ*g/ml iFMDV for 72 h. Supernatants were analyzed using ELISA as described previously [30]. Briefly, plates were coated with a mAb against interferon-gamma (IFN-*γ*) (kindly donated by Dr. L. Babiuk). Samples and recombinant IFN-*γ* standard (Serotec, UK) were added, and IFN was detected using rabbit polyclonal anti-IFN-*γ* antibodies. After incubation, biotinylated goat anti-rabbit IgG antibody was added and then HRP-conjugated streptavidin (KPL, USA) was added. The plates were washed, incubated with (OPD)-H_2_O_2_, and read at 492 nm. The IFN-*γ* concentration was calculated from interpolation of data in the standard curve.

### 2.9. ELISPOT Assay for FMDV-Specific ASC

Mononuclear cell (MNC) suspensions were obtained from lymphoid tissues as previously described [31]. A FMDV-ASC ELISPOT assay was developed for this study. Ninety-six-well nitrocellulose plates (Millipore, MA) were coated overnight with 2.4 *μ*g/well inactivated purified FMDV O1/Campos/Bra/58 and blocked with 4% skim milk for 1 h at room temperature (RT). MNC were seeded in FMDV-coated plates in 2-fold dilutions (2.5 × 10^5^ and 1.25 × 10^5^ cells per well) in triplicate, and wells were incubated overnight at 37°C with 5% CO_2_. After 5 washes with phosphate-buffered saline (PBS), mouse anti-bovine IgG1 or IgG2 monoclonal antibodies (BD-Serotec, Oxford, UK) were added (1 : 500 dilution) and incubated for 1 h at RT. Reactions were revealed with anti-mouse IgG (HRP)-labeled conjugate (KPL, UK) for 1 h at RT, followed by the addition of TrueBlue peroxidase substrate (KPL, UK). IgM and IgA ASC were detected with HRP-labeled sheep anti-bovine IgM and IgA sera (Bethyl), diluted 1 : 5.000, and revealed as described above. Spots corresponding to ASC were visualized and counted manually under a stereomicroscope. Spots from control wells were subtracted from experimental wells, and results were expressed, unless otherwise indicated, as the mean number of ASC per 1 × 10^6^ cells for triplicate wells.

### 2.10. Statistical Analysis

The InfoStat program was used. One-way analysis of variance (ANOVA) and posttests were used to compare data between three or more groups.

## 3. Results

### 3.1. B_2_T and B_4_T Induce Anti-Peptide and FMDV-Specific Antibodies in Cattle

**Figure 2:**
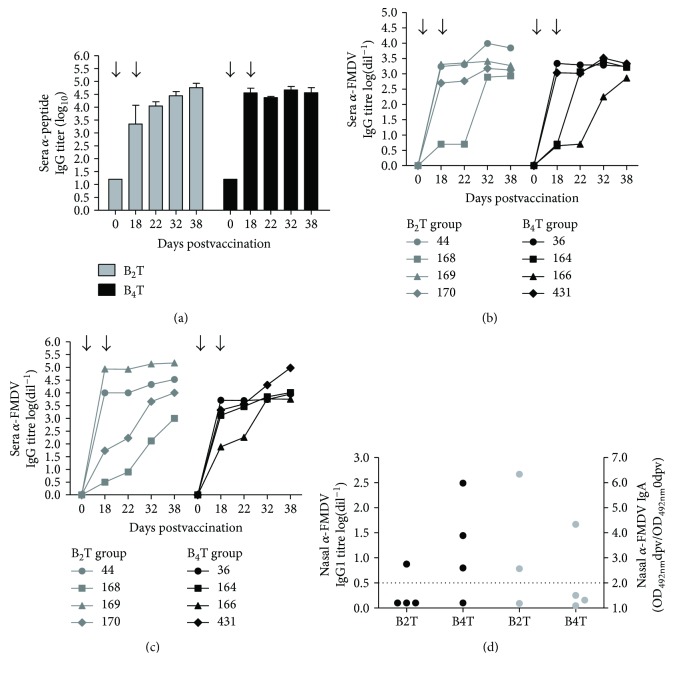
Antibody detection by ELISA in vaccinated cattle. Animals were immunized on days 0 and 18 (arrow) with B_2_T or B_4_T vaccine. (a) Kinetics of anti-peptide serum antibodies. Bars represent the mean IgG titres from bovines in each group (gray, B_2_T; black, B_4_T) throughout the experiment (error marks, SD). (b, c) Kinetics of total IgG and IgG1 anti-FMDV O1/Campos/Bra/58 serum antibodies. Titres were calculated as log_10_ of the last reciprocal dilution above cut-off. Data points represent the IgG titre (b) or IgG1 titre (c) from each animal represented by different shapes (right legend) throughout the experiment. (d) FMDV-specific mucosal IgG1 and IgA responses. Nasal swabs were collected at 22 dpv. Each point represents the nasal IgG1 anti-FMDV antibody titres (log_10_) (black) or IgA (gray) anti-FMDV O1/Campos/Bra/58 antibody level of each animal. The cut-off was established as the mean value of mock-vaccinated animals plus twice the SD value (dotted line).

At 38 dpv, all animals inoculated with either B_2_T or B_4_T constructs developed specific and pronounced anti-peptide (Figure 2(a)) as well as anti-FMDV total IgG (Figure 2(b)) and IgG1 responses (Figure 2(c)).

At 38 dpv, high anti-FMDV IgG titres were detected in all animals with an average titre of 3.4 ± 0.4 and 3.3 ± 0.3 in B_2_T and B_4_T groups, respectively (Figure 2(b)). However, some animals showed a significant increase in IgG titre only after the second peptide dose (168, 164, and 166), while the others were able to achieve high IgG titres since the first immunization. Lastly, the results showed that the anti-FMDV IgG1 was the predominant isotype in all vaccinated animals given that there was a minor difference between total IgG and IgG1, and low levels of specific FMDV IgG2 were detected in B_2_T and B_4_T groups with average antibody titres of 1.1 ± 0.3 and 1.9 ± 0.7, respectively (data not shown).

### 3.2. FMDV-Specific Mucosal Immunity

Animals from the B_4_T group exhibited high levels of anti-FMDV IgG1 in nasal secretions at 22 dpv, with the exception of bovine 166; however, at this time, animals from the B_2_T group did not present high anti-FMDV IgG1 titres (Figure 2(d)). When IgA was measured in nasal secretions, animals 44 and 170 in the B_2_T group and 36 in the B_4_T group showed positive anti-FMDV IgA levels, indicating that these peptide constructs were able to induce not only systemic but also local mucosal immunity.

### 3.3. Analysis of the Neutralizing Capacity of the Sera

The VNT against the homologous virus O/UKG/11/2001 were determined at 32 dpv, and average values of 1.2 ± 0.3 and 1.3 ± 0.3 were found in the B_2_T and B_4_T groups, respectively (Table 1). Although the VNT against the heterologous type O virus (O1/Campos/Bra/58) was in the limit of the detection threshold, a log_10_ neutralization index with values of 1.3 ± 0.5 and 1.8 ± 0.7 could be determined for sera from animals of the B_2_T and B_4_T groups, respectively (Table 1). As expected, no NI values were found in the preimmune sera (*T* = 0). The log_10_ NI of sera from 4 bovines vaccinated with commercial vaccine was 2.0 ± 0.3 (data not shown).

### 3.4. Specific Cellular Immune Response and IFN-*γ* Release in Vaccinated Animals

Before challenge, at 32 dpv, specific *in vitro* lymphoproliferations were conducted using different stimuli. Significant values of proliferation (SI ≥ 2) to the peptide used for immunization (B_2_T or B_4_T) were found in 2 out of 4 animals of the B_2_T group and in 3 out of 4 animals of the B_4_T group (Table 2(a)). Responses to dendrimers not used for immunization were similar to those achieved with the immunizing peptide while the number of animals that recognized the T-cell peptide alone was lower. In the B_2_T group, PBMC from bovine 44 significantly proliferated in response to the T peptide, and animals 168 and 169 showed no response to any stimulus. In the B_4_T group, only cells from bovine 36 proliferated when stimulated with the T peptide. PBMC from negative control animals (Table 2(a)) and from all bovines at day 0 did not respond to any peptide (data not shown). In the group immunized with the commercial vaccine, 3 animals out of 5 showed positive proliferation against iFMDV and 1 out of 5 against both dendrimers (Table 2(a)).

The levels of IFN-*γ* secreted in vitro by PBMC from immunized animals were also determined at 32 dpv (Table 2(b)). Positive IFN-*γ* responses to the immunizing peptide were found in 3 out of 4 animals of both the B_2_T and B_4_T groups, and the responses were similar to those induced by the dendrimers not used for immunization. In the B_2_T group, only cells from bovine 44 secreted IFN-*γ* when they were stimulated with the T peptide, whereas 3 out of 4 animals of the B_4_T group secreted IFN-*γ* even without stimulus (Table 2(b)).

On the other hand, bovines 169 and 166 did not secrete IFN-*γ* and were considered as nonresponders. PBMC from negative control animals (Table 2(b)) and from all bovines at day 0 (data not shown) did not respond to any peptide.

### 3.5. Different Clinical Score Protection after Challenge

Since the aim of the study was to investigate the protection afforded by the dendrimeric peptides and the infection with FMDV type O other than O1/Campos/Bra/58 was not possible at INTA, bovines were challenged with this virus, an experimental design that allows the assessment of the cross-protection conferred by the dendrimers. Thus, all animals were challenged at 44 dpv by nasal instillation with infective FMDV O1/Campos/Bra/58, and protection was measured by monitoring clinical signs in animals after the challenge. As shown in Table 3, the two negative control animals showed typical FMDV lesions, while, remarkably, bovines 44 (from the B_2_T group) and 36 (from the B_4_T group) did not show any lesions on their feet along the 7 days of clinical observation and were considered as PPG, albeit they showed a single vesicle in the tongue at 7 dpc. In addition, animals 170 (from the B_2_T group) and 431 (from the B_4_T group) showed a delay in the onset of symptoms (PP) during the normal course of disease, and the lesions in the feet appeared on 7 dpc, while those in the mock-vaccinated animals appeared on 3 or 4 dpc. Bovines 168, 169, 164, and 166 were nonprotected (NP); they presented vesicle in the tongue, mouth, and feet. At 7 dpc, all animals showed lesions in their mouth or tongue (Table 3).

### 3.6. Mucosal Adaptive Antibody Responses in Peptide-Vaccinated Cattle after Nasal Infection

**Figure 3:**
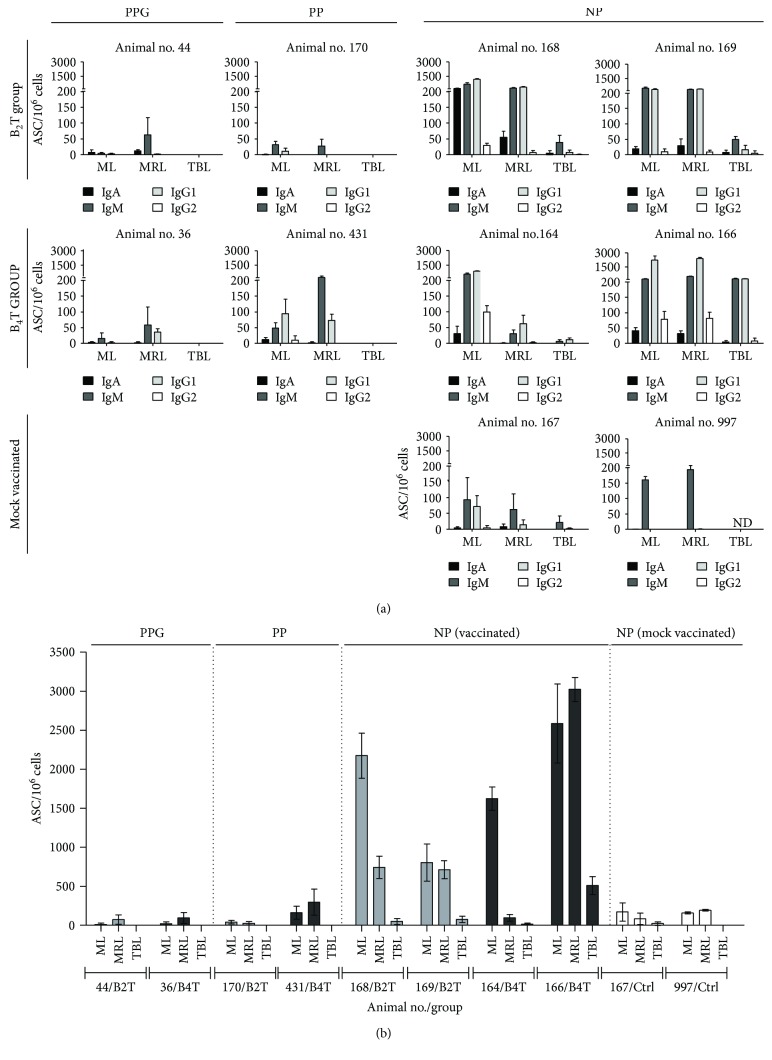
Profiles of the FMDV-ASC detected in B_2_T- and B_4_T-vaccinated cattle after FMDV challenge. (a) Mononuclear cells were purified from mandibular lymph nodes (ML), medial retropharyngeal lymph nodes (MRL), and tracheobronchial lymph nodes (TBL) and characterized by the FMDV-ASC ELISPOT assay, using monoclonal (IgG1 and IgG2) or polyclonal (IgM and IgA) antibodies against bovine immunoglobulin isotypes as probes. (b) Total FMDV-ASC in ML, MRL, or TBL. Results are expressed as the mean number of FMDV-specific ASC per 1 × 10^6^ extracted cells. Each bar represents the mean value of 3 replicates ± SD. PPG: protected against podal generalization; PP: partial protected; NP: non-protected.

Animals were euthanized at 7 dpc, and the FMDV-specific mucosal immune responses were studied along the respiratory tract by means of a FMDV-ASC ELISPOT assay (FMDV-ASC ELISPOT). The results showed three profiles of responses (Figure 3(a)) according to the degree of protection (PPG, PP, or NP) observed in the animals. In general, PPG and PP bovines showed a very low number of ASC in mandibular lymph nodes (ML) and medial retropharyngeal lymph nodes (MRL), with the exception of bovine 431. Tracheobronchial lymph nodes (TBL) of animals 44 and 170 from the B_2_T group (PPG and PP, resp.) and animals 36 and 431 from the B_4_T group (PPG and PP, resp.) did not show secretory cells producing FMDV-specific antibodies (Figures 3(a) and 3(b)). When ASC from peptide-immunized NP animals were studied, IgM and IgG1 were the dominant isotypes of antibody detected in ML; high amounts of IgA ASC (>200 ASC/10^6^ cells) were detected in animal 168. However, the other NP bovines (169, 164, and 166) presented a low number of IgA ASC (<50 ASC/10^6^ cells). On the other hand, IgG2 ASC were detectable in ML at this time with values 10- to 80-fold lower than the IgG1 value. Finally, in vaccinated NP animals, high amounts of IgM and IgG1 ASC were detected in MRL (excluding bovine 164). Finally, animals 168, 169, and 166 presented a high level of total ASC in ML and MRL (Figure 3(b)). NP vaccinated (168,169, 164, and 166) and mock-vaccinated animals (167 and 997) also showed responses in TBL at the lower respiratory tract. IgM and IgG1 antibodies against FMDV were the isotypes secreted.

Animal 166, which showed delayed humoral response against virus, presented the highest number of IgG1 ASC (>2 × 10^3^ ASC/10^6^ cells) in ML and MRL. Concordantly, bovine 166 was the only animal that showed high numbers of IgM and IgG1 ASC in TBL.

In mock-vaccinated animals (167 and 997), ML and MRL were the most stimulated secondary lymphoid organs at 7 dpc, IgM was the dominant isotype among the FMDV-ASC developed in these organs. In animal 167, IgG1 was the next isotype with regard to the detection level, with levels 10- to 30-fold lower than those detected in the ML of NP vaccinated bovines. When the total FMDV-ASC was calculated, PPG and PP animals presented very low numbers of ASC in comparison with NP vaccinated animals (Figure 3(b)).

### 3.7. IFN-*γ* Secretion by Mononuclear Cells (3 Days Post-FMDV Challenge)

**Figure 4:**
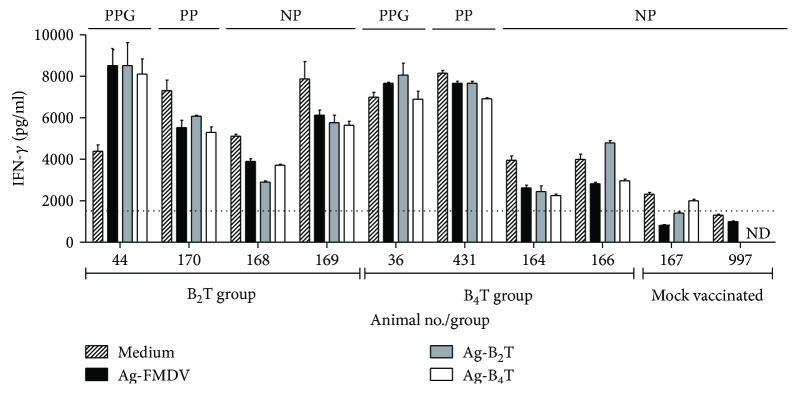
IFN-*γ* production by PBMC from peptide-vaccinated bovines after challenge. PBMC were purified at 3 dpc and cultured in the presence of peptide or inactivated virus. The supernatants were tested by a sandwich ELISA. Results are expressed in pg/ml by interpolation in a cytokine standard curve. Each bar represents the mean value of 2 replicates of supernatants ± SD. PPG: protected against podal generalization; PP: partial protected; NP: nonprotected.

In order to determine the memory immunity induced in vaccinated animals after the challenge with the live virus, the level of IFN-*γ* secreted in vitro by mononuclear cells of those animals was measured (Figure 4). At 3 dpc, PPG animals (44 and 36) presented IFN-*γ* levels between 8 and 9.5 × 10^3^ pg/ml in the supernatant of PBMC stimulated with iFMDV, B_2_T, or B_4_T peptides. On the other hand, PP animals (170 and 431) showed high levels of IFN-*γ* even without stimuli as also observed in PPG animal 36. Significant differences were found in ASC, ML, and MRL of PPG and PP animals compared to NP-vaccinated cattles. Surprisingly, bovine 169 presented high levels of IFN-*γ*.

## 4. Discussion

Synthetic peptides corresponding to the protective B- and T-cell epitopes can be considered good candidates for FMD vaccines as, among other advantages, they are safe and support a rational design and their production and characterization are simple. The development of successful peptide vaccines has been limited for a number of reasons, including those associated with “in vivo” stability, poor immunogenicity of linear peptides, and lack of adequate T-cell activation due to MHC polymorphism of the host species [32, 33]. Previous results in pigs vaccinated with B_2_T or B_4_T dendrimeric peptides allow concluding that multiple presentation of the B-cell epitope is advantageous over a simple juxtaposition of the epitopes for the induction of humoral and cellular immune responses [34]. Recently, Blanco et al. [16] reported that 100% of pigs vaccinated with B_2_T dendrimeric peptides bearing type O FMDV O/UKG/11/2001 sequences of a B- (VP1 136–154) and a T-cell epitope (3A 21–35) were protected after the challenge with homologous FMDV. In this report, we have explored the immunogenicity of B_2_T and B_4_T dendrimers in cattle and showed that they can elicit cross-reactive immune responses against a heterologous type O strain, FMDV O1/Campos/Bra/58, including partial protection to challenge.

In our experiment, specific antibody responses to virus were observed in all cattle receiving peptide vaccines; however, even when neutralizing antibodies against FMDV O/UKG/11/2001 were detected, their levels were lower than those found in pigs immunized with the same peptides.

The amino acid sequence of the B-cell epitope VP1 (136–154) between FMDV O/UKG/11/2001 and O1/Campos/Bra/58, the virus used for cattle challenge, differs in 3 amino acids. Nevertheless, Wang et al. [10] reported that pigs vaccinated with a peptide containing a consensus type O VP1 sequence (residues 129–169) from 75 historic and prevalent isolates (including O1/Campos/Bra/58 and O/UKG/11/2001) and a promiscuous artificial Th site, developed humoral immunity against FMDV O1/Campos/Bra/58. Indeed, the neutralizing activity against FMDV O1/Campos/Bra/58 was detected in our peptide-immunized cattle, albeit the magnitudes of the responses were lower than those elicited against FMDV O/UKG/11/2001. B_2_T and B_4_T peptides induce neutralizing antibodies against FMDV O/UKG 11 at low levels, but they do not neutralize FMDV O1 Campos (100 DITC50).

Despite the presence of anti-peptide and anti-FMDV antibodies in sera, they may not have the affinity necessary to effectively neutralize the virus, and only 25% of the B_2_T- or B_4_T-vaccinated animals were PPG after the challenge with FMDV O1/Campos/Bra/58. It is possible that when using another adjuvant or other amounts of peptides in the vaccine, the immune response could increase in cattle, achieving the maturation of the antibodies affinity for the viral neutralization of FMDV O1/Campos/Bra/58.

The isotype of antibodies elicited in cattle by the two dendrimers differs from those induced in swine [16]. Pigs vaccinated with B_2_T showed a trend towards increased levels of specific IgG1 and IgG2 relative to pigs vaccinated with B_4_T. In contrast, here B_2_T- or B_4_T-vaccinated bovines elicit levels of IgG1 higher than those of IgG2. These changes seem to reflect marked differences in how the immune systems of swine and cattle recognize and process the dendrimeric immunogens. In any case, it is noteworthy that the same nomenclature for subclasses among different species often leads to the misleading belief that these subclasses are homologous and have the same functions.

Animal-to-animal variation is found in the protective responses evoked by peptide vaccines, including those against FMDV [13, 35], which has been associated with the MHC-restricted recognition of T-cell epitopes included in their composition. On the other hand, the different immune responses against peptide might be indicative of marked differences in the recognition of T epitopes between cattle and swine. The T-cell peptide 3A (21–35) was well studied in pigs [6] but not in cattle, and our results support that T epitope 3A (21–35) is not recognized by the majority of bovines.

Our findings suggest that in some instances, animals showing the highest immunological parameters measured against peptides and iFMDV were better protected against viral challenge. Bovines 44 (B_2_T) and 36 (B_4_T) elicited high levels of antibodies against virus (although animal 36 showed levels of neutralizing antibodies of 1.2) and developed high levels of IgA specific against virus in nasal secretions as well as a positive lymphoproliferative response not only against dendrimeric peptides but also against the epitope T 3A (21–40). All these positive parameters in bovines 44 and 36 correlated with a protective immune response. Indeed, these were the only two PPG animals. On the other hand, and despite at the time of challenge the level of antibodies (measured by ELISA) being similarly high for all cattle, animals that showed modest humoral response initially (at 18 or 22 dpv) failed to be protected against viral challenge, which may be due to the lack of antibody maturation. Nonprotected animals 168 (B_2_T) and 164 (B_4_T) showed antibody responses against iFMDV only after receiving a second dose of vaccine, and their viral neutralization titres were lower than 1.2 (VNT positive for FMDV ≥ 1.2 [27]). These results suggest that although neutralizing antibodies are important in protecting against viral challenge, other factors could also favor protection.

Cattle are highly susceptible to FMDV, and the virus usually gains entry through the respiratory tract of these animals [36]. Moreover, FMDV replicates in tissues of the upper respiratory system [37, 38], the soft palate and pharynx being preferential sites of FMDV replication and persistence in ruminants. A feature of the mucosal system in ruminants is the prominence of IgG1 relative to IgA in nasal secretions.

The study of antibody responses in local lymphoid tissues indicates that the systemic FMD vaccination of cattle with dendrimeric peptides can effectively promote the presence of anti-FMDV ASC in lymphoid tissues associated with the respiratory tract. In addition, the detection of both FMDV O1/Campos/Bra/58-specific ASC and antibodies following vaccination shows that these peptides, encompassing FMDV O/UKG/11/2001 sequences, were able to induce a cross-reactive ASC response.

In peptide-vaccinated unprotected animals, viral challenge by nasal instillation triggered an antibody response compatible with a local anamnestic recall upon contact with replicating FMDV, suggesting that peptide vaccination might induce the circulation of virus-specific B-lymphocytes, including memory B-cells that differentiate into ASC soon after contact with the infective virus. Thus, NP animals showed a strong stimulation of FMDV-specific B-lymphocytes to locally produce antibodies all along the respiratory tract, including in the tracheobronchial lymph nodes (TBL) with frequencies of ASC much higher than those in mock-vaccinated infected animals. In the NP animals, ASC were detected in all studied organs, and the isotype of the antibodies (mainly IgM and IgG1) revealed that even when B_2_T and B_4_T peptides elicited specific memory B-cells, the response failed to stop the advance of the challenge virus. Conversely, in peptide-vaccinated PPG and PP animals, no FMDV-ASC were detected in TBL possibly because the virus did not reach that area. Thus, in animals 44 and 36 (PPG) and 170 and 431 (PP), cells producing antibodies against FMDV were not found in TBL, and in general the total number of ASC induced was low.

It has been proposed that structural features lend FMDV capsids towards stimulating B-cells in a T-independent manner [39, 40] and acute cytopathic viral infections can result in the accelerated induction of antibody in a T-independent manner [41, 42], providing a rapid means of stopping the systemic spread of the virus [43]. In the absence of CD4+ T-cells, cattle can produce class-switched antibody rapidly in response to the FMDV challenge [40], and a rapid induction of FMDV-specific plasma cells has been also reported in local lymphoid tissue following live-virus exposure, which, again, is consistent with a T-independent response [21]. In this report, at 7 dpc, limited amounts of IgM and IgG1 ASC were detected in ML, MRL, and TBL of one of the negative control animals (167), and only IgM was found in the other (997), a result that is consistent with a primary response against FMDV. A greater increase in the number of ASC of isotypes IgA, IgM, IgG1, and IgG2 was found by Monso et al. [20] at 6 dpc in ML, TBL, and MRL; this discrepancy may be related with the difference in the viral dose and inoculation route employed by these authors (10^7^ TCID50, aerosol) compared with those of our study (10^4^ BID50, nasal instillation).

Overall, our results support that immunization in cattle with dendrimeric peptides B_2_T and B_4_T can elicit humoral and cellular immune responses and confer partial protection against a heterologous virus challenge that is associated with the induction of solid T-cell responses as well as of an anamnestic antibody response. Experiments are in progress to address whether modifications such as the replacement of the T-cell peptide by one widely recognized by cattle can result in an improvement of the protective response elicited by these dendrimeric peptides.

## Figures and Tables

**Table 1:** Virus neutralizing titres prechallenge.

Group	Animal no.	Neutralizing antibodies (38 dpv)	
		VNT^a^ O/UK/01	log_10_ NI O1/C
B_2_T	44	1.60	2.0
	170	1.20	1.3
	168	1.10	0.8
	169	0.90	1.0

B_4_T	36	1.20	1.3
	431	1.75	2.7
	164	1.10	2.0
	166	1.20	1.3

Commercial vaccine	522	—	1.8
	800	—	2.1
	809	—	1.6
	810	—	2.3
	820	—	2.8

Negative controls	167	<0.9	0.0
	997	<0.9	0.3

^a^Titre of virus-neutralizing antibody at day 38 post vaccination. O/UK/01: FMDV O/UKG/11/2001; O1/C: FMDV O1/Campos/Bra/58.

**(a):** 

Group	Animal no.	SI (cpm Ag/cpm medium)			
		Ag-B_2_T	Ag-B_4_T	Ag-T	iFMDV
B_2_T	44	56.0	39.6	4.0	1.1
	170	3.0	5.2	1.0	1.2
	168	1.4	1.8	1.1	1.1
	169	2.0	1.9	0.9	1.5

B_4_T	36	2.5	3.6	2.3	1.4
	431	3.5	1.6	1.0	1.2
	164	4.2	7.3	1.4	1.8
	166	4.0	5.7	0.8	1.3

Commercial vaccine	522	2.9	2.5	2.0	3.2
	800	1.4	2.0	1.2	1.6
	809	1.5	1.9	1.2	4.1
	810	1.2	1.4	0.9	0.9
	820	0.7	0.7	0.7	2.8

Negative	167	1.0	0.7	1.0	1.0
controls	**997**	1.0	1.4	0.9	0.9

**(b):** 

Group	Animal no.	IFN-*γ* (× 10^2^ pg/ml)				
		Medium	Ag-B_2_T	Ag-B_4_T	Ag-T	iFMDV
B_2_T	44	7.8	57.8	76.2	61.4	7.1
	170	14.0	32.1	30.2	12.4	11.3
	168	19.3	17.3	13.2	11.8	13.3
	169	12.3	14.0	14.6	15.0	17.9

B_4_T	36	28.7	36.0	16.0	27.9	28.1
	431	32.8	15.7	54.0	34.0	35.0
	164	33.9	24.2	40.0	31.2	37.8
	166	7.9	6.0	6.5	6.8	6.8

Commercial vaccine	522	51.6	30.4	19.8	11.6	36.1
	800	7.4	7.6	7.4	7.1	7.5
	809	24.3	44.2	36.1	25.8	44.2
	810	16.9	14.3	13.7	18.0	15.9
	820	7.3	7.3	6.5	9.4	6.9

Negative controls	167	8.4	5.2	6.5	7.2	6.1
	997	9.6	12.0	14.0	13.3	14.8

(a) Lymphoproliferation of PBMC from vaccinated cattle (32 dpv) determined by ^3^H-thymidine incorporation. Results were expressed as SI. PBMC were stimulated *in vitro* following incubation with dendrimeric peptides B_4_T, B_2_T or epitope T, iFMDV O1/Campos/Bra/58, or medium alone. Radioactivity was measured with b-scintillation counter. SI was calculated as cpm of each antigen specific proliferation Ag/cpm of cells basal proliferation. SI values ≥ 2.5 are considered positive. (b) IFN-*γ* production by PBMC after peptide stimulation as in (a). Supernatants were tested by ELISA, and the results, expressed in pg/ml, were calculated by interpolation in a cytokine standard curve. For each peptide, the cut-off was calculated as the mean IFN-*γ* production of PBMC from animals at day 0 plus 2 SD (≥15.0 × 10^2^ pg/ml). Positive IFN-*γ* productions above cut-off are underlined.

**Table 3:** Clinical scores of vaccinated cattle after challenge.

Group	Animal no.	Clinical score (dpc)^a^				Protection^b^
		2 dpc	3 dpc	4 dpc	**7 dpc**	
B_2_T	44	0	0	0	**2**	PPG
	170	0	0	0	**5**	PP
	168	0	0	5	**6**	NP
	169	0	0	3	**6**	NP

B_4_T	36	0	0	0	2	PPG
	431	0	0	0	**5**	PP
	164	0	0	3	**5**	NP
	166	0	5	6	**6**	NP

Negative controls	167	0	0	4	6	NP
	997	0	4	4	6	NP

^a^Clinical score was established after the challenge and was determined by the number of feet presenting FMD lesions plus the presence of vesicles in the snout and/or mouth, 6 being the maximum score. ^b^Animals with no lesions on the feet were PPG. Animals with a delay in the onset of symptoms of disease were PP, and animals with lesions on their feet before 7 dpc were considered NP.

## Additional Points

*Highlights.* Dendrimeric peptides containing T and B epitopes were designed as vaccine candidates against FMDV for cattle. Dendrimeric peptides evoke in immunized cattle heterologous cellular and humoral immune responses. Tetravalent and bivalent presentation of B-cell epitopes linked to the T epitope conferred partial heterologous protection in cattle.
